# Is COVID-19 to Blame?

**DOI:** 10.22074/cellj.2020.7564

**Published:** 2020-09-08

**Authors:** Alireza Asgari

**Affiliations:** Aerospace Medicine Research Center, Aja University of Medical Sciences, Tehran, Iran

**Keywords:** Communicable, Convergence, COVID-19, Infectious, Non-Communicable, SARS-CoV-2

## Abstract

Mankind is witnessing economic uncertainty due to a health crisis as never before. In the era of industrialization where the emergence of invisible enemies of humans is causing a great death toll, "nothing seems more universal than health", the old proverb in nearly all human cultures is once again rebirthed by the current COVID-19 pandemic. Nevertheless what is distinctive is that the SARS-CoV-2 seems to be unequally targeting a particular sector of the populations with risk factors for preventable diseases. Comorbidities, mainly non-communicable diseases (NCDs), seem to be the primary contributors of the current pandemic and not the SARS-CoV-2 per se. The present letter attempts to underscore the converging pattern of communicable (CDs) and NCDs in human toll. For the tens of thousands of lives coming to an end since the turn of the year, we are all truly sad, but thankful to the virus for unearthing the grave need of the mankind to improve his life style and behaviors. It directs us to revisit the values and ultimately save millions of lives in future.

## Introduction

In the era of industrialization and globalization where the emergence of invisible enemies of humans is growing at a speedy pace with inevitable consequences, nothing seems more universal than health The first 5-page situation report of WHO on 21^st^ of January 2020 announced that it was not until 7^th^ of January 2020 that Chinese scientists identified the new coronavirus as being the cause of their latest 44 cases of pneumonia (1). The SARS-CoV-2 seems to be selecting the older stratum of the world populations where the risk of preventable death is high. Comorbidities, mainly noncommunicable diseases (NCDs), seem to be the primary contributors of the current pandemic and not the SARS-CoV-2 *per se*. The present commentary attempts to underscore the converging pattern of communicable (CDs) and NCDs in *human toll,* where only a few frequently asked questions are barely answered, even if asked from the most knowledgeable subject matter experts in the field.

The first United Nation High-Level Meeting (UN HLM) on Health issues was held in 2001 for HIV and yet millions are awaiting a real breakthrough amid tremendous progress in research and management of AIDS. The second HLM was held in 2011 where the impact of NCDs on countries economic growth was brought to surface. Cost of illness, whether direct or indirect, economic loss of productivity and the value of statistical life were the three approaches used to calculate the burden by a collaboration between WHO and the Harvard School of Public Health in 2011 (2). The alarm was substantiated with compelling evidence: more than 60% of all deaths is caused by NCDs which will affect economic growth, more so in low- and middle-income countries. The report anticipated the cumulative cost of NCDs in 2030 to be about 31 trillion USD, by utilizing the burden of NCDs in 2010 and mathematically projecting it into 2030. The EPIC model of WHO, introduced by Abegunde and Stanciole (3), simulated labor and capital features of the economy as two main productive factors that were linked to economic growth. It was concluded by proposing: "a unified front is needed to turn the tide on NCDs". Nearly a decade has passed and yet solid evidence is needed to substantiate whether the world has taken that report seriously in tackling NCDs and if the commitments at national level were scaled to the severity of the threat, despite published doubts (4). The economic catastrophe that inevitably follows the NCDs is felt strongly by the next generation. That is how wide apart the health and economics aspects of NCDs are!

On the same track, the latest UN HLM was convened in September 2019 on Universal Health Coverage issue with the objective of achieving and promoting physical and mental health and well-being of all world inhabitants, leaving no one behind. To cognize the thoughts cloud of the participants, a few keywords were searched for in the published list of pre-registered statements from multistakeholder constituency/institutions in that convention (5), including "non-communicable", "communicable"and "infectious". The frequencies of their appearance were 6:1:1, respectively. Presuming that the frequency of words can stand for the level of focus, concern and mindfulness, it seems as if infectious diseases were not at the center of the focus. A few days passed, however, and the whole world was caught by surprise by coronavirus, for the third time in recent decades, but only this time surpassing the thresholds of a pandemic event in less than a couple of months. Now the COVID-19 has created a real dramatic picture. Since the early days of the current pandemic, human death has tolled to 138,008 by 16^th^ Apr 2020 (JHU corona map) in nearly 4 months, worldwide (6). The worst may yet to come, God forbid, but even then it does not match deaths of NCDs during the same period, more than 200K (7, 8). In a single-centered retrospective study done in Wuhan, China, 50% of nonsurvivors suffered from chronic diseases (9). Another study by Zhou and colleagues from a pneumonia hospital in Wuhan reported that 67% of non-survivors had comorbidities, hypertension in the first place followed by diabetes (10). The picture is more or less the same in Italy (11) and probably everywhere. The virus has hit and shaken the world badly, but only this time in a unique and unprecedented manner: the *health impact* and the *economic turmoil* are felt simultaneously. *Unhealthiness* and death concurrent with economic distress, leading to immediate unemployment and cash shortages, let aside the health access disparities, travel restrictions, voluntary quarantines, and forced lock-downs, all occurred in about 2 short months!

Following the striking report on the burden of NCDs (2) many countries came to become knowledgeable that economic threats do not necessarily stem from miscalculating megatrends in the emerging markets and/ or industries, global recession, disputes and disagreements between oil producing countries, and not even from climate change. However, knowledge *per se* would not bring any change, it needs to be enacted. The number of preventable deaths is still on the rise as if the due interventions are themselves suffering from inefficiency and chronicity. The total number of deaths from NCDs between 2007 and 2017 increased by 27% and that of total YLDs rose more than 50% (4). It seems that humans are not good at responding to events with very slow time course change. Biological principles dictate that if a stimulus intensity rises in several minutes instead of a few seconds, our senses will probably not detect it and the stimulus may easily be ignored, regardless of the absolute magnitude at most times. In fact, we tend to be more vigilant to kinetics of the stimuli rather than their absolute magnitude. As such, we have never advised our family members to eat quality food and engage in physical activity as often as we urge them to wash their hands nowadays, mainly for one reason: we do not see them fall sick and die in a matter of days if they suffer from nutritional inefficacies, hypertension or diabetes. The chronicity of NCDs seems to be the factor that is making all the differences. The chronicity of NCDs renders country health authorities to pass by in silence. The chronicity allows WHO not to declare a state of worldwide emergency.

### Convergence of infectious and preventable diseases

It was the general belief of the past century that most infectious diseases are acute illnesses and chronic diseases are mostly non-communicable (12). However, with growing evidence, scientists now believe that a significant portion of chronic diseases are associated with infections. However, the two artificial fields of CDs and NCDs that scientists have long before dichotomized for simplicity are proving insufficient. They are potentiating one another in favor of greater illnesses of humans. Today, pandemic of infectious diseases and epidemics of NCDs are converging on a global scale, a notion that Marias et al. (13). intelligently anticipated to be escalating in the coming decades. COVID-19 is clearly displaying that message, if nothing else. No matter how wild the COVID-19 evolves to become across various world epigenome in the remaining months of 2020, it will not cost humans lives as much as NCDs. To be prepared for future infectious pandemics, the share of NCDs in economic burden of COVID-19 is to be determined and appropriate flags raised. It seems that NCDs, in particular cardiovascular diseases, are the key player in the current crisis. They should once again be stressed to be put at the center of the business sectors’ radar in all countries.

Let us focus on the fact that the COVID-19 is not causing all the problems *per se,* it is the comorbidity of the infected that has created all the chaos and confusion. SARS-CoV-2 does not seem to be as cruel as flu virus. As of to date and according to the limited information about its mechanistic path in human body, the comorbidities are killing the host and NOT the COVID-19. If the poor virus had an official speaker and a podium to be questioned by aggressive reporters, it would have pleaded "NOT GUILTY"! Most probably, what the virus is trying to unearth is our vulnerability, predominantly caused by NCDs. It is forcing us to realize that it is our way of living that is backfiring. In other words, SARS-CoV-2 is doing a good job of inversing the chronic character of NCDs into an acute picture, visible and experienced by nearly all individuals across borders.

In the 20^th^ century, influenza and measles, as singlepathogen diseases, were defeated when NCDs were either absent or at their infancy and not hitting all-time records. However, COVID-19 has picked a time to spread where NCDs are at their highest and human microbiome diversity at their lowest (14, 15). Dynamics of COVID-19 behavior in an already health-compromised human body may need a shift in the focus of scientist struggling in the front line of the current viral invasion. In that sense, one potential research priority of delving into the unexpected human toll of the COVID-19 pandemic, is to find a qualitative and quantitative relationship between the clinical outcome of COVID-19 with the type and chronicity of co-existing morbidities and the corresponding microbiome diversity. This approach may partially heal the fragmented studies of epidemiology in different populations. The fact is buried in the tangled web of information in hundreds of hospitals now managing the patients. When the dynamics of the COVID-19 pandemic and its synergies with the epidemics of NCDs, meet the threshold of an official and WHO-tagged *syndemic, *the metaphor of "the blind man and the elephant" may get bridged in order to improve nations’ resilience.

As David Jones puts it: "The history of epidemics offers considerable advice, but only if people know the history and respond with wisdom" (16). Can we take a lesson from the present pandemic to prevent, detect, and mitigate chronic diseases and to reduce probability of future viral epidemics, even pandemics? The "viral security" that the world is running after may not be attained without "NCD security" and even other unknown health "securities". Charles Rosenberg states: "epidemics start at a moment in time, proceed on a stage limited in space and duration, follow a plot line of increasing revelatory tension, move to a crisis of individual and collective character, then drift toward closure" (17). As Jones claims in his recent perspective: "societies and their citizens misunderstand the relative importance of the health risks they face. Citizens and their leaders need to think carefully, weigh risks in context, and pursue policies commensurate with the magnitude of the threat" (16). We either need to win or learn the current pandemic, but we cannot afford to lose it.
